# Reliability and Feasibility of Change of Direction Testing in National Basketball Players

**DOI:** 10.5114/jhk/200868

**Published:** 2025-04-30

**Authors:** Tom Faulks, Adam Petway, Mark Drury, Sibi Walter

**Affiliations:** 1 Faculty of Health, University of Canterbury, Christchurch, New Zealand.; 2 Human Factors & Athlete Engineering, Center for Advanced Vehicular Systems, Mississippi State University, Mississippi, USA.

**Keywords:** exercise, functional performance, physical fitness, muscle strength

## Abstract

The reliability and feasibility of a 2-2 shuffle test time against on-court lateral change of direction performance was examined. Ten male national league players performed two trials of the 2-2 shuffle test per direction and were compared against their total steals throughout the season. Intraday reliability of the test was computed using Bland-Altman plots, intraclass correlation coefficients (ICCs) and coefficients of variations (CVs). Anthropometric and total steals differences between fast and slow 2-2 shuffle performance were assessed with an independent t-test, percent difference (Diff %), and effect size (ES). The 2-2 shuffle test time for dominant (ICC = 0.86, CV = 8.61%) and non-dominant (ICC = 0.90, CV = 9.30%) directions met pre-determined reliability thresholds (ICC > 0.8, CV < 10%). Faster 2-2 shufflers were significantly shorter (1.91 ± 0.03 vs. 2.02 ± 0.09, Diff % −5.45, p = 0.03, ES = −1.3) in height and accrued more total steals than their slower counterparts (15.60 ± 9.24 vs. 9.00 ± 6.44, Diff % 42.31, p = 0.22, ES = −0.8). Practitioners may add the 2-2 shuffle to their assessment battery as the test time is a reliable metric and can show the direction for on-court lateral performance as reflected by total steals.

## Introduction

Change of direction (COD) ability is a key performance indicator in many sports, but not the least in basketball, during which players repeatedly accelerate and decelerate in three-dimensional time and space (Abdelkrim et al., 2007; Ferioli et al., 2020; Petway et al., 2020a; Stojanović et al., 2018). For defensive basketball in particular, the ability to efficiently accelerate and decelerate laterally is paramount for contending with competition demands (Leidersdorf et al., 2022; Schelling et al., 2016; Shimokochi et al., 2013). In fact, the frequency of lateral shuffling was much higher in basketball when compared to other sports, with 313−446 lateral shuffles per competition (Taylor et al., 2017). Further research into the activity demands of basketball competition highlights that up to 31% of live game actions are comprised of lateral shuffling movements (McInnes et al., 1995). Here, players must be quick enough to stay in front of the ball-handler to contain dribble penetration and contest passing or shooting, but also be strong enough to absorb force during player-to-player collisions (Morrison et al., 2022; Petway et al., 2022; Shimokochi et al., 2013). This can happen during defensive ball screen possessions, for example, challenging the defensive players ability to translate their mass laterally and occupy space while maintaining stability with the contact incurred from the screener. As such, within the sporting activity of basketball, the ability to expresses lateral COD ability could have implications on both technical and tactical advantages, particularly on defence. It is for this reason that the assessment of lateral COD ability from shuffling may provide strength and conditioning coaches with prognostic/diagnostic information (Faulks et al., 2024b) to prescribe interventions, monitor training effects, and measure outcomes of interest for defensive basketball situations. Lateral COD ability, for the interpretation of this study, specifically refers to the overarching skills and abilities expressed to change direction laterally while shuffling.

The T-test is a frequently used COD test among basketball players (Morrison et al., 2022; Wen et al., 2018) and can well differentiate between player performance levels (Abdelkrim et al., 2010a), along with a strong linear sprint speed (r = −0.92 to −0.83) (Poole et al., 2017) correlation to performance time. Unfortunately, the side shuffle distances do not replicate basketball match play movements (Bourgeois et al., 2017; Leidersdorf et al., 2022; Morrison et al., 2022). A shorter-distance T-test has been developed to replicate high-intensity, multidirectional demands specific to basketball (Scanlan et al., 2021; Wen et al., 2018), but this test may not specifically indicate lateral COD ability (Abdelkrim et al., 2010b; Scanlan et al., 2011), due to the linear sprint component masking the player’s ability to decelerate and accelerate laterally (Nimphius et al., 2018). To this end, COD tests isolated to lateral shuffling over distances that resemble match play activity may provide a more ecologically valid transfer between the test and the competitive skill (Bourgeois et al., 2017; Nimphius et al., 2018; Zukowski et al., 2023). As such, the capacity of a lateral COD test to accurately measure on-court lateral performance may be dependent on the degree to which the testing procedures can encompass physical abilities and skills required for competition.

Thus far, the kinetic and kinematic variables that are characteristic of faster lateral COD performance have been investigated in basketball players (Faulks et al., 2024a; Leidersdorf et al., 2022; Shimokochi et al., 2013), but the link between the ability to move in the frontal plane and on-court lateral performance has yet to be determined. While the ability to move effectively in the frontal plane plays a pivotal role in occupying space defensively, choosing an appropriate, meaningful variable to represent on-court lateral performance is still nebulous within the sport of basketball. The reason for such difficulty is that on-court metrics are influenced by factors tactically that may not have implications on the ability to move laterally. For example, defensive win shares, i.e., the credit attributed to players based on their ability to prevent opposing teams from scoring, may seem like an appropriate way to understand how players express their lateral COD ability during competition, but it is influenced by a myriad of factors outside of the physical ability and skill of the athlete (Guo et al., 2022; Kobat, 2018). As such, total steals may be a better proxy to rank the ability to move laterally in competition because it is less affected by teammates and the quality of opponents, however, the usefulness of this statistic according to lateral COD ability has yet to be investigated. Furthermore, no previous research has examined the test-retest reliability of a lateral COD test in basketball players. If proved to be a reliable and feasible measure, the use of a lateral COD test seems warranted for basketball player performance analysis to identify predictors of performance in defensive situations and guide decision making for training interventions based on the findings. Therefore, the purpose of this research was twofold: (1) to examine the intraday reliability of the time obtained during the lateral COD test amongst New Zealand National Basketball League (NZNBL) players, and (2) to investigate the feasibility of the 2-2 shuffle test to show direction for on-court lateral performance via total steals.

## Methods

### Experimental Approach to the Problem

This retrospective cohort study was performed during the competitive period of the 2023 NZNBL season. Lateral COD data from the 2-2 shuffle test was collected in June 2023 by the strength and conditioning staff using an intraday design to establish the test-retest reliability. Each player completed one submaximal and two maximal 2-2 shuffles per direction. Total steals statistics were collected throughout the competitive season, allowing the use of analysis to examine the differences between 2-2 shuffle time and on-court lateral performance in fast and slow performers using a median split analysis.

### Participants

Ten male NZNBL players (5 guards, 5 forwards, 23.2 ± 2.6 years, 1.97 ± 0.1 m; 95.3 ± 11.6 kg) from the same team volunteered to participate in this study. The recruited players were free from any medical condition, exercise disorder or injury that might compromise performance in 2-2 shuffle at the time of testing. Written informed consent was obtained from all players before participating in the study. Testing was conducted in the player's training facility on an indoor basketball court surface, and participants wore their own basketball shoes during the testing session. The University of Canterbury Human Research Ethics Committee approved the experimental protocol (approval code: HREC 2023/23/LR; approval date: 22 May 2023).

### Procedures

#### 2-2 Shuffle Test

Anthropometric measures of players were recorded before testing. After partaking in a standardized warm-up, testing was conducted. For the side shuffle test, each player started with one submaximal familiarisation trial and two maximal performances in each direction on their own volition. Each lateral shuffle was performed with maximum effort from the start line to the COD line, then players braked the lateral movement with one foot crossing the COD line and quickly shuffling laterally back to the start/finish line. The distance between the start/finish line and COD was 2.5 m. From the start/finish line the timing gates (Smart Speed; Fusion Sport, Brisbane, Australia) were placed at 50 cm and at a height of 40 cm to minimise any early start trigger. The tester provided verbal encouragement to the players for giving their maximum effort and quickest time in each trial and the performance time was recorded. Between each trial a 10-min rest interval was provided. If any running or feet crossing was observed in a player, then the trial was not recorded, and was repeated after rest. For the reliability analysis the first and the second test time were used, and the quickest trial times for dominant and non-dominant sides were averaged and used in the median split analysis with anthropometric and total steals data.

#### On-Court Lateral Performance Data

Total steals statistics were collected from an online publicly available database (RealGM, 2024). All available total steals statistics were retrieved for each player that completed the 2-2 shuffle test. Hence every participant had two scores to be included in the analysis, total steals and 2-2 shuffle time.

### Statistical Analysis

Intraday reliability was reported using the intraclass correlation coefficient (ICC), the coefficient of variation (CV), and confidence intervals (CI). ICC ≥0.7, CV ≤10% and ICC ≥0.8, CV ≤5% were considered to reveal acceptable and good reliability, respectively (Weir et al., 2020; Zukowski et al., 2023). The Bland-Altman plots were used to represent the bias (mean difference) and 95% confidence limits between trials. Participants were stratified as “fast” or “slow” based on a median split analysis of 2-2 shuffle performance (Leidersdorf et al., 2022; Scanlan et al., 2021). The differences between groups were reported using an independent *t*-test to determine whether the two groups differed from each other in anthropometric factors and total steals between fast and slow 2-2 shuffle performers. In addition to the statistical significance (i.e., *p*-value), we focused on the effect sizes and percent differences to determine the directionality and practical significance of the differences when interpreting the results due to the small sample size. Thresholds for effect sizes were evaluated as suggested by Cohen (2013) and Weir et al. (2020): small (*d* = 0.20–0.49), medium (*d* = 0.50–0.79), and large (*d* ≥ 0.80). As previously mentioned, the best repetitions of the 2-2 shuffle per direction were averaged and used for subsequent analysis. By averaging the data set, we therefore assumed the uncertainty about estimating the averages were consistent because the measurements of the athletes were taken from the same sample structure. Mean, standard deviation (SD), percent difference, and Cohen’s *d* effect size utilising the pooled SD were calculated for each variable. Data were analysed using R Studio (Version 2023.12.1+402) with the alpha set at 0.05 for the threshold for significance.

## Results

**Figure 1:**
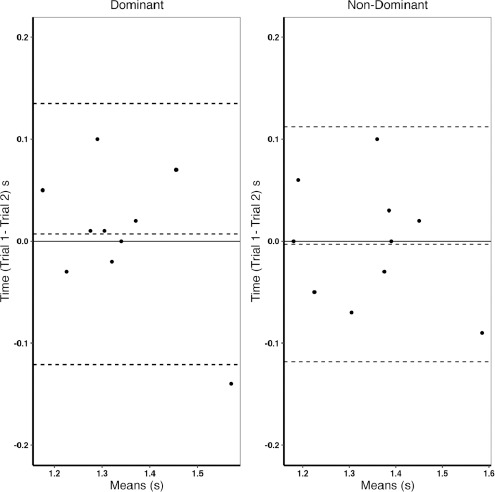
Difference in mean trials (trial 1− trial 2) vs. 2-2 shuffle means Bland-Altman plots of the dominant and non-dominant sides, dashed lines indicate bias and 95% limits of agreement; s = seconds

The 2-2 shuffle test time for both directions exhibited the ICC ≥ 0.8 and the CV ≤ 10% with ICC 95% CIs between 0.52 and 0.98, as detailed in Table 1. The Bland-Altman plots revealed a small mean offset lying above (2-2 shuffle dominant) and below (2-2 shuffle non-dominant) zero with 1 of the 20 points lying outside the upper-lower 95% CI (Figure 1). Participants stratified as fast demonstrated significantly lower 2-2 shuffle time (1.23 ± 0.05 s vs. 1.40 ± 0.07 s, % Diff = −12.14%, *p* ≤ 0.01, ES = −1.58) compared to participants stratified as slow. The *t*-test results comparing anthropometric measures and total steals differences between fast and slow players are presented in Table 2. There was a significant difference in height (1.91 ± 0.03 m vs. 2.02 ± 0.09 m, % Diff = −5.45%, *p* = 0.03, ES = −1.30) and a large effect size for total steals (15.60 ± 9.24 vs. 9.00 ± 6.44, % Diff = −42.31%, *p* = 0.22, ES = 0.80) between fast and slow participants.

**Table 1:** Test-retest reliability of 2-2 shuffle performance.

2-2 Shuffle Side	Trial 1	Trial 2	CV	ICC (95% CI)
Dominant (s)	1.34 ± 0.10	1.33 ± 0.13	8.61%	0.86 (0.52−0.96)
Non-dominant (s)	1.34 ± 0.12	1.35 ± 0.14	9.30%	0.90 (0.66−0.98)

s = seconds; CV = coefficient of variation; ICC = intraclass correlation coefficient; 95 % CI = 95% confidence interval

**Table 2:** Anthropometric, 2-2 shuffle, and total steals differences between male National Basketball League players with fast and slow lateral change of direction times (Mean ± SD).

Variables	Fast (n = 5)	Slow (n = 5)	% Diff	ES	*p*-value
AVG 2-2 Shuffle (s)	1.23 ± 0.05	1.40 ± 0.07	12.14%	−1.58	< 0.01*
Total Steals	15.60 ± 9.24	9.00 ± 6.44	42.31%	0.80	0.22
Body Mass (kg)	96.44 ± 14.76	94.20 ± 9.14	2.32%	0.19	0.78
Height (m)	1.91 ± 0.03	2.02 ± 0.09	5.45%	−1.30	0.03*

AVG = average; % Diff = percentage difference; ES = effect size; * = statistical significance

## Discussion

The purpose of this study was to examine the intraday reliability of the 2-2 shuffle test, as well as to assess the feasibility of the 2-2 shuffle test and show the direction for on-court lateral performance via total steals. We established acceptable to good intraday reliability in the 2-2 shuffle for dominant and non-dominant sides. We also found that faster performers in the 2-2 shuffle test were significantly shorter and accrued more total steals to their slower counterparts. These results could have corresponding implications for informing future off-court testing batteries for basketball players.

Although an isolated lateral COD assessment has been used previously in the literature (Leidersdorf et al., 2022; Shimokochi et al., 2013), this is the first study to investigate the intraday reliability of test time during a lateral COD task in NZNBL players. The main finding was that the 2-2 shuffle test time showed acceptable to good intraday reliability (ICC = 0.86−0.90, CV = 8.61−9.30%) for dominant and non-dominant sides. This is comparable to research by Stojanovic et al. (2019), which reported acceptable to good intraday reliability in the lane agility test (ICC = 0.88, CV = 7.3%) amongst adolescent male basketball players. Whilst the reliability of these metrics can be deemed ‘acceptable’ to ‘good’, based on the 95% CIs of the ICC (0.52−0.98), the level of reliability should be regarded as moderate to excellent (Koo et al., 2016). Furthermore, the percentage change required to be confident that a real change has occurred rather than an inside error of the test, as indicated by CVs, may be too high for some contexts. For example, a CV of < 5% may be deemed acceptable for use within a high-performance setting to have confidence in detecting small changes in performance. Thus, our conclusion is that test time as measured during the 2-2 shuffle test is reliable, but it is not overly sensitive to small changes in performance, which are important considerations to successfully integrate a test into practice. It therefore may be prudent that practitioners considering implementing the 2-2 shuffle test perform a familiarisation session prior to any formal data collection to improve the reliability and establish in-house thresholds for meaningful changes in performance using their own testing equipment.

Considering test reliability, performance tests that indicate how they translate into performance in competition are highly relevant to practitioners (Zukowski et al., 2023). This notion highlights the limitation of current practice in assessing COD ability as the data describing this interaction between performance in tests and outcomes of interest in basketball are scant (Lombard et al., 2024). Thus, the secondary purpose of this study was to investigate the feasibility of the 2-2 shuffle to differentiate between players and show direction in on-court lateral performance via total steals. To the best of the authors’ knowledge, this is a novel concept in that no study has examined how lateral COD performance is linked with success in defensive basketball situations as quantified by total steals. The median split analysis showed that faster performers in the 2-2 shuffle accumulated more steals in competition (15.60 ± 9.24 vs. 9.00 ± 6.44, % Diff = −42.31%, *p* = 0.22, ES = 0.80) compared to their slower counterparts. However, faster performers in the 2-2 shuffle also tended to be shorter (1.91 ± 0.03 m vs. 2.02 ± 0.09 m, % Diff = −5.45%, *p* = 0.03, ES = −1.30). There could be a few explanations for these findings: (1) shorter players have shorter levers which may provide a mechanical advantage to displace laterally and occupy space than taller, longer legged players due to the angular impulse-momentum principle; (2) three of the five players stratified as fast were guards who by nature are going to have shorter levers; and (3) guards had more of an opportunity to get steals based on the demands of the position within the context of the sport. Interestingly, a non-significant difference was established between body mass in fast and slow 2-2 shuffle performers. However, there is notable variance within the dataset as indicated by the SD and one must consider that shorter players resultantly have their leg mass located closer to the hip joint despite having comparable system mass, permitting the leg to move at faster velocities, all things being equal. While these explanations do not capture the full scope of an athlete’s capability to effectively translate their mass laterally, we believe that it does at least in-part explain why the present findings have been established.

We believe the above are important considerations when interpreting the results, but it is important to recognise that the 2-2 shuffle is not intended to be an all-encompassing test of the qualities required to excel in defensive situations, nor are total steals capable of representing the ‘total picture’ for on-court lateral performance within the context of defensive basketball. The factors that impact test and on-court performance are too vast, dynamic, and complex. It follows that our findings serve a broader purpose which is to gain insights into the commonalities amongst faster performers in the 2-2 shuffle and to indicate direction in a small population. Thus, given the magnitude of difference between the unique constructs, the results are likely meaningful enough to justify the use of the 2-2 shuffle test in basketball performance and rehabilitation settings to help explain the ability to move laterally, both on and off the court. From a practical standpoint, the 2-2 shuffle is also time efficient, generally non-fatiguing, simple to perform, and requires minimal technology. These considerations have major implications on the critical thought process for the feasibility of implementing the 2-2 shuffle test in the applied environment.

This study has some limitations which should be acknowledged. Firstly, it is important to be mindful of the small sample size, which may have impacted the statistical analyses and, as a result, the findings here may only be generalizable to the sample recruited. However, all recruited participants were active players on a professional roster in New Zealand, Australia, and Europe, illustrating the exceptionality of the sample investigated. In addition to this, it is worth noting that this investigation was conducted during the competitive season, which imposes complexities and rigors of schedule constraints, media obligations, and travel demands, owing to the realities of applied research (Petway et al., 2020b; Zukowski et al., 2023). Secondly, participants wore their own basketball shoes, presumably with different friction coefficients between the shoe and the court surface. However, since no slipping was observed, it can be assumed that the maximal frictional forces they received were below the maximal static friction force. Thus, wearing their own basketball shoes was thought to minimally affect shuffle time. Finally, we did not account for factors that may have had an impact on total steals, such as player experience, individual technical-tactical aspects, wingspan, reach, total minutes played, and fatigue levels. Testing had to fit into the players congested competition schedule.

## Conclusions

Our results suggest that the 2-2 shuffle protocols used in this study were reliable and helped explain on-court defensive performance as reflected by total steals. Although there are a multitude of COD tests reported in the literature, this study should aid practitioners in decision making for whether the inclusion of the 2-2 shuffle is warranted or not when designing an assessment battery for basketball players. Future research should dissect the capacity of the 2-2 shuffle to explain on-court lateral performance using specific in-game situations to gain additional insights and clues into commonalities amongst the game’s best lateral movers.

## References

[ref1] Abdelkrim, B., Castagna, C., Jabri, I., Battikh, T., El Fazaa, S., & El Ati, J. (2010a). Activity profile and physiological requirements of junior elite basketball players in relation to aerobic-anaerobic fitness. Journal of Strength & Conditioning Research, 24(9), 2330−2342. 10.1519/JSC.0b013e3181e381c1 20802281

[ref2] Abdelkrim, B., Chaouachi, A., Chamari, K., Chtara, M., & Castagna, C. (2010b). Positional role and competitive-level differences in elite-level menʼs basketball players. Journal of Strength & Conditioning Research, 24(5), 1346−1355. 10.1519/JSC.0b013e3181cf7510 20393355

[ref3] Abdelkrim, B., Fazaa, N., El, & Ati, E. J. (2007). Time–motion analysis and physiological data of elite under-19 year-old basketball players during competition. British Journal of Sports Medicine, 41(2), 69−75. 10.1136/bjsm.2006.032318 17138630 PMC2658931

[ref4] Bourgeois, F., McGuigan, M., Gill, N., & Gamble, G. (2017). Physical characteristics and performance in change of direction tasks: A brief review and training considerations. Journal of Australian Strength & Conditioning, 25, 104−117.

[ref5] Cohen, J. (2013). Statistical power analysis for the behavioral sciences. Academic Press.

[ref6] Ferioli, D., Schelling, X., Bosio, A., La Torre, A., Rucco, D., & Rampinini, E. (2020). Match activities in basketball games: Comparison between different competitive levels. Journal of Strength & Conditioning Research, 34(1), 172−182. doi: 10.1519/JSC.0000000000003039 30741861

[ref7] Faulks, T., Drury, M., & Walter, S. (2024a). Reliability and relationship between hip muscle strength and change of direction performance among basketball players. International Journal of Kinesiology and Sports Science, 12(3), 29−36. 10.7575/10.7575/aiac.ijkss.v.12n.3p.29

[ref8] Faulks, T., Sansone, P., & Walter, S. (2024b). A systematic review of lower limb strength tests used in elite basketball. Sports, 12(9), 262. 10.3390/sports12090262 39330739 PMC11435599

[ref9] Guo, T., Cui, Y., Min, W., Zhang, W., Mi, J., & Shen, Y. (2022). Exploring the relationship between basketball rotation and competitive performance using substitution network analysis. Journal of Sports Sciences, 40(24), 2704−2713. 10.1080/02640414.2023.2189216 36895098

[ref10] Kobat, L. R. (2018). *Win Shares & Rookie Contracts in the NBA* University of South Dakota. https://red.library.usd.edu/honors-thesis/14; accessed on 22 May 2023.

[ref11] Koo, T. K., & Li, M. Y. (2016). A guideline of selecting and reporting intraclass correlation coefficients for reliability research. Journal of Chiropractic Medicine, 15(2), 155−163. doi: 10.1016/j.jcm.2017.10.001. 27330520 PMC4913118

[ref12] Leidersdorf, E., Rauch, J., Reeves, T., Borkan, L., Francis, J., Storey, L., Souza, E. O. D., Elliott, M., & Ugrinowitsch, C. (2022). Reliability and effectiveness of a lateral countermovement jump for stratifying shuffling performance amongst elite basketball players. Sports, 10(11), 186. 10.3390/sports10110186 36422955 PMC9697629

[ref13] Lombard, W. P., & Lambert, M. I. (2024). Physical fitness metrics and their relationship to locomotor activity profiles among female international field hockey players across an Olympic cycle. Journal of Science & Medicine in Sport, 27(5), 341−353. 10.1016/j.jsams.2024.02.003 38431456

[ref14] McInnes, S. E., Carlson, J. S., Jones, C. J., & McKenna, M. J. (1995). The physiological load imposed on basketball players during competition. Journal of Sports Sciences, 13(5), 387. 10.1080/02640419508732254 8558625

[ref15] Morrison, M., Martin, D. T., Talpey, S., Scanlan, A. T., Delaney, J., Halson, S. L., & Weakley, J. (2022). A systematic review on fitness testing in adult male basketball players: Tests adopted, characteristics reported and recommendations for practice. Sports Medicine, 52(7), 1491−1532. 10.1007/s40279-021-01626-3 35119683 PMC9213321

[ref16] Nimphius, S., Callaghan, S. J., Bezodis, N. E., & Lockie, R. G. (2018). Change of direction and agility tests: Challenging our current measures of performance. Strength & Conditioning Journal, 40(1), 26−38. 10.1519/SSC.0000000000000309

[ref17] Petway, A. J., Freitas, T. T., Calleja-González, J., Medina Leal, D., & Alcaraz, P. E. (2020a). Training load and match-play demands in basketball based on competition level: A systematic review. PloS One, 15(3), e0229212−e0229212. 10.1371/journal.pone.0229212 32134965 PMC7058381

[ref18] Petway, A. J., Freitas, T. T., Calleja-González, J., Torres-Ronda, L., & Alcaraz, P. E. (2020b). Seasonal variations in game activity profiles and players' neuromuscular performance in collegiate division i basketball: Non-conference vs. Conference tournament.Frontiers in Sports & Active Living, 2, 592705. 10.3389/fspor.2020.592705 PMC7739638 33345170

[ref19] Petway, A. J., & Richman, R. (2022). Basketball Mechanics. Ultimate Athlete Concepts.

[ref20] Poole, J., Fox, J., & Scanlan, A. (2017). The contribution of linear sprinting and lateral shuffling to change of direction T-test performance in semi-professional, male basketball players. Journal of Australian Strength & Conditioning, 25(3), 6−12.

[ref21] RealGM. https://basketball.realgm.com/international/league/75/New-Zealand-NBL; accessed on 22 May 2023.

[ref22] Scanlan, A., Dascombe, B., & Reaburn, P. (2011a). A comparison of the activity demands of elite and sub-elite Australian men's basketball competition. Journal of Sports Sciences, 29(11), 1153−1160. 10.1080/02640414.2011.582509 21777151

[ref23] Scanlan, A. T., Wen, N., Pyne, D. B., Stojanovic, E., Milanovic, Z., Conte, D., Vaquera, A., & Dalbo, V. J. (2021). Power-related determinants of modified agility t-test performance in male adolescent basketball players. Journal of Strength & Conditioning Research, 35(8), 2248−2254. 10.1519/JSC.0000000000003131 30893280

[ref24] Schelling, X., & Torres-Ronda, L. (2016). An integrative approach to strength and neuromuscular power training for basketball. Strength & Conditioning Journal, 38(3), 72−80. doi: 10.1519/SSC.0000000000000219

[ref25] Shimokochi, Y., Ide, D., Kokubu, M., & Nakaoji, T. (2013). Relationships among performance of lateral cutting maneuver from lateral sliding and hip extension and abduction motions, ground reaction force, and body center of mass height. Journal of Strength & Conditioning Research, 27(7), 1851−1860. 10.1519/JSC.0b013e3182764945 23085969

[ref26] Stojanovic, E., Aksovic, N., Stojiljkovic, N., Stankovic, R., Scanlan, A. T., & Milanovic, Z. (2019). Reliability, usefulness, and factorial validity of change-of-direction speed tests in adolescent basketball players. Journal of Strength & Conditioning Research, 33(11), 3162−3173. 10.1519/JSC.0000000000002666 29927890

[ref27] Stojanović, E., Stojiljković, N., Scanlan, A. T., Dalbo, V. J., Berkelmans, D. M., & Milanović, Z. (2018). The activity demands and physiological responses encountered during basketball match-play: A systematic review. Sports Medicine, 48, 111−135. 10.1007/s40279-017-0794-z 29039018

[ref28] Taylor, J. B., Wright, A. A., Dischiavi, S. L., Townsend, M. A., & Marmon, A. R. (2017). Activity demands during multi-directional team sports: a systematic review. Sports Medicine, 47, 2533−2551. doi: 10.1007/s40279-017-0772-5 28801751

[ref29] Weir, J. P., & Vincent, W. J. (2020). *Statistics in Kinesiology* (4th ed.). Human Kinetics.

[ref30] Wen, N., Dalbo, V. J., Burgos, B., Pyne, D. B., & Scanlan, A. T. (2018). Power testing in basketball: Current practice and future recommendations. Journal of Strength & Conditioning Research, 32(9), 2677−2691. 10.1519/JSC.0000000000002459 29401204

[ref31] Zukowski, M., Herzog, W., & Jordan, M. J. (2023). Single leg lateral and horizontal loaded jump testing: Reliability and correlation with long track sprint speed skating performance. Journal of Strength & Conditioning Research, 37(11), 2251−2259. 10.1519/JSC.0000000000004533 37235211

